# Transcription Regulation of E-Cadherin by Zinc Finger E-Box Binding Homeobox Proteins in Solid Tumors

**DOI:** 10.1155/2014/921564

**Published:** 2014-08-13

**Authors:** Thian-Sze Wong, Wei Gao, Jimmy Yu-Wai Chan

**Affiliations:** Department of Surgery, The University of Hong Kong, Pokfulam, Hong Kong

## Abstract

Downregulation of E-cadherin in solid tumors with regional migration and systematic metastasis is well recognized. In view of its significance in tumorigenesis and solid cancer progression, studies on the regulatory mechanisms are important for the development of target treatment and prediction of clinical behavior for cancer patients. The vertebrate zinc finger E-box binding homeobox (ZEB) protein family comprises 2 major members: ZEB1 and ZEB2. Both contain the motif for specific binding to multiple enhancer boxes (E-boxes) located within the short-range transcription regulatory regions of the E-cadherin gene. Binding of ZEB1 and ZEB2 to the spaced E-cadherin E-boxes has been implicated in the regulation of E-cadherin expression in multiple human cancers. The widespread functions of ZEB proteins in human malignancies indicate their significance. Given the significance of E-cadherin in the solid tumors, a deeper understanding of the functional role of ZEB proteins in solid tumors could provide insights in the design of target therapy against the migratory nature of solid cancers.

## 1. Introduction

Epithelial cadherin (E-cadherin, cadherin type 1, CD324, or CDH1) is involved in the cell cohesiveness and assembly of identical or different cell types during tissue construction and morphogenesis [1]. E-cadherin functions as adhesion molecule at adherens junctions and binds cells through homophilic interactions (i.e., E-cadherin on one cell binds to another E-cadherin molecule on the neighboring cell) in a Ca^2+^-depending manner. Removal of the calcium ion from the extracellular environment will disrupt the homophilic interactions between E-cadherin molecules, loose the contact between adjacent cells, and promote degradation. Precise transcriptional control of E-cadherin gene expression is essential during developmental reprogramming, cellular differentiation, and cancer progression [2, 3]. Further, E-cadherin suppression enhances the development of migratory and invasive phenotype by increasing cell motility and facilitating dissociation from the surrounding extracellular matrix of the primary site. Hence, exploiting the fundamental processes involved in E-cadherin suppression is thought to have a significant implication in the context of cancer prevention and migration inhibition.

Destruction of the cadherin-cadherin adhesion linkages at the cell junction is the initial step for cell dissociation and detachment. In solid cancers, cancer cells prompt to change the degree of cell adhesiveness in order to disseminate from the primary cancer site by altering E-cadherin expression [4]. Hereby cancer cells could acquire the mesenchymal phenotype which facilitates them to invade into the surrounding tissues and through basement membranes [5]. This process is referred to epithelial-mesenchymal transition (EMT) and the motile mesenchymal-like cells are characterized by repression of epithelial-associated genes and expression of filopodia and lamellipodia [6]. With the advances in molecular technique, it is now recognized that timely and precise control of E-cadherin expression plays a pivotal role in the molecular reprogramming during EMT and is closely linked with cancer aggressiveness.

## 2. Enhancer Box (E-Box) at the Promoter Region of E-Cadherin Encoding Gene

Transcription factors could bind to the cis-regulatory elements in the promoter region of eukaryotic genes [7]. The 5′ proximal promoter regions of E-cadherin gene contain GC-rich sequence, palindromic sequence E-pal, and E-boxes which allows direct binding of specific transcription regulators [8–11]. Although the transcription regulation mechanism of E-cadherin in cancer cells is not fully elucidated, emerging evidence suggested that coordinated recruitment of different transcription factors/repressors to the promoter region plays a key role in controlling timely expression of E-cadherin in different developmental stages. Enhancer box or E-box motifs (5′-CAnnTG-3′) are palindromic sequence elements which are the binding sites of basic helix-loop-helix (bHLH) class of DNA-binding transcription factors [12, 13]. Using serial 5′ deletion constructs and mutated constructs containing E-cadherin promoter, it has been demonstrated that there are at least 2 E-box elements present in the promoter region with the essential role in controlling E-cadherin expression in both mouse and human genome [11, 14]. Theoretically, the binding of transcription activators or repressors to the E-boxes of the E-cadherin gene could control gene expression at transcription level by allowing the binding of coregulatory proteins.

## 3. Zinc Finger E-Box Binding Homeobox (ZEB) Protein Family

Binding of ZEB1 and ZEB2 to the E-cadherin E-boxes has been implicated in the regulation of E-cadherin expression in multiple human cancers [15]. ZEB proteins are sequence-specific DNA-binding transcription factors. In upper vertebrates, the ZEB belongs to the zfh family comprising ZEB1 (deltaEF1) and ZEB2 (Smad-interacting protein 1, SIP1) [16]. The zhf family members are characterized by the characteristic flanking zinc finger clusters and homeodomain-like domain in their protein with specific DNA-binding ability [17, 18]. ZEB1 and ZEB2 contain the helix-loop-helix motif allowing them to bind to the bipartite E-boxes within the E-cadherin promoter region with high specificity [3]. Controlled expression of ZEB protein is critical based on the fact that ZEB null mice will die shortly after birth [19]. In normal tissues, expression of ZEB1 and ZEB2 is observed in tissues undergoing differentiation such as T cell differentiation and skeletal differentiation [20, 21]. In addition, expression of ZEB proteins is discriminative between cancers with different grading and cancer types [22–25]. ZEB expression is partly controlled by epigenetic mechanisms based on the observation that the transcriptional functions of ZEB are responsive to the HDAC inhibitor Trichostatin A [26]. By recruiting different coactivators or corepressors, ZEB proteins can perform different functions in the context of chromatin remodeling [3, 27]. With the recruitment of C-terminal binding protein CtBP, ZEB proteins function as transcription repressors [28]. CtBP1 could interact with histone deacetylase to attenuate gene expression by targeting the promoter region [29]. CtBP2 could interact with the ZEB proteins through the three PLDLS-like motifs and mediate transcription suppression [30]. In the presence of CtBP1/2, transcription repression effect was remarkable increased [31]. However, it should be noted that CtBPs binding to ZEB protein is not always necessary in the ZEB-mediated transcription attenuation [32]. Sumoylation (addition of ubiquitin-like modifier SUMO to the lysine residues) of ZEB protein at Lys391 and Lys866 by the polycomb protein Pc2 could alleviate the E-cadherin repression mediated by ZEB proteins [33]. Recent findings suggested that ZEB expression is controlled by the microRNA which targets the ZEB mRNA transcripts [34–36]. In addition, ZEB could control the microRNA expression by interfering the microRNA promoter activity forming a reciprocal feedback loop in controlling EMT [37]. At present, the mechanism for ZEB to switch from transcription repressors to activators remains poorly understood. In oligodendrocytes, Sip1 can activate Smad7 transcription and modulate various developmental stages [38]. Further, it has been demonstrated that ZEB2 can form complexes with the coactivators p300 and pCAF (p300/CBP associated factor) [39]. Evolutionary functional analysis on vertebrate ZEB protein suggested that the ZEB protein contains functional CtBP-interacting domain, Smad-binding domain, homeodomain, and sumoylation sites which could possibly be the potential sites for its regulation [40].

Dysregulation of ZEB1/2 and E-cadherin has been involved in diverse tumorigenic processes resulting in the development of mesenchymal phenotype, stem-like cell character, resistance to therapeutic agents, aggressiveness during EMT, adaptive stages under hypoxic microenvironment, and cancer progression. Given the significance of E-cadherin in the solid tumors, a deeper understanding of the properties of ZEB proteins is critical. Here, we reviewed the current evidence on transcription regulation of E-cadherin by ZEB1 and ZEB2 proteins in solid tumors.

## 4. Bladder and Renal Cancer

Downregulation of E-cadherin has been implicated in the migration and invasion of bladder cancer cells [41]. In clinical specimens, reduced E-cadherin expression accompanied with increased CD10 (a membrane-bound zinc-dependent metalloprotease) expression is observed in both transitional cell carcinoma and squamous cell carcinoma [42]. In addition, the specific association of E-cadherin reduction with urothelial cell carcinoma leading to the suggestion that loss of E-cadherin is responsible for the progression, invasion, and metastasis of cancer cells derived from the transitional epithelium [42]. In plasmacytoid urothelial carcinoma, complete loss of E-cadherin in the cell membrane is found in more than 70% and nuclear accumulation is detected in 48% of the patients [43]. In urothelial carcinoma, E-cadherin level is an indicator of poor prognosis with linking to tumor recurrence and disease-free survival rates [44]. Methylation analysis showing that promoter DNA hypermethylation is a major contributor, which attenuates transcription activity of the E-cadherin gene. With the use of meta-analysis, it is shown that E-cadherin hypermethylation in bladder cancer was prevalent in the Asian populations in comparison with the Caucasian populations [45]. Although membrane E-cadherin is frequently lost in the tumor cells, soluble E-cadherin could be detected in the urine of bladder cancer patients and is correlated with the tumor size and lymph node metastasis [46].

ZEB dysregulation is involved in the TGF-*β*1-induced EMT in renal tubular epithelial cancer cells and is closely associated with the microRNA-200 family [47, 48]. ZEB1 expression is higher in the high-grade urothelial carcinoma in comparison with the low-grade counterparts [24]. In contrast, ZEB2 expression is significantly higher in infiltrating carcinoma than high-grade urothelial carcinoma [24]. It has been suggested that ZEB1 expression is regulated by nuclear *β*-catenin upon stimulation [49]. In bladder cancer, *β*-catenin signaling cascades can be activated by various routes. In which, many evidence pointed to the glycogen synthase kinase 3*β*- (GSK3*β*-) ZEB1 cascade which was triggered through phosphatidylinositol 3-kinase (PI3 K)/Akt pathways [49–51]. Further, noncoding RNA including microRNA-23b and long noncoding RNA MALAT-1 has also been suggested to be the transcriptional regulator of ZEB1 and ZEB2 in bladder cancers [52, 53].

## 5. Brain Cancer

In intracranial cancers, glioblastoma is the most common form [54]. At present, there is still no effective curative treatment for malignant glioblastoma and the survival time is <1 year upon diagnosis. The 5-year survival rate is less than 5% if the cancer is treated with radiotherapy alone [55]. Although E-cadherin suppression is observed in the brain cancer tissues, the functions of E-cadherin in the tumor cells remain to be verified as another cadherin member, and N-cadherin seems to plays a more significant role in brain cancer aggressiveness. Low E-cadherin expression is found in most of the glioblastoma tissues and is associated with the differentiation status of the glioblastoma [56, 57]. In comparison with the tumor tissues, E-cadherin expression is rare in the glioblastoma cell lines [58, 59]. Locking down E-cadherin expression in the E-cadherin expression glioma cells will have a negative impact on cell proliferation and migration [59]. It has been suggested that E-cadherin plays a different role in the glioblastoma tissues (in comparison with the epithelial cancers) based on the observation that E-cadherin expression in the glioblastoma could possibly be associated with the poor clinical outcomes [59]. At present, little is known about the regulatory mechanism of E-cadherin in brain cancer. In medulloblastoma, the methylation frequency of E-cadherin gene was not high (8%) [60]. In the context of ZEB suppression, binding of ZEB1 to the E-cadherin promoter was dependent on the activation of NF-*κ*B in glioblastoma [61]. In the glioblastoma cell lines, it has been demonstrated that high ZEB2 levels could suppress E-cadherin, thereby regulating cancer cell differentiation [62].

## 6. Breast Cancer

Loss of E-cadherin is characterized in the aggressive breast cancers including aggressive lobular carcinoma and lobular carcinoma* in situ* in comparison with the less invasive tumor type such as ductal cancers [63]. This led to the suggestion that E-cadherin is involved in mediating tumor progression and metastasis in the breast cancers. Three E-box elements have been suggested to be involved in E-cadherin silencing [9]. It has been shown that the ZEB1 expression is upregulated by steroid hormones such as progesterone [64]. A subpopulation of breast cancer cells with CD44+/CD24− phenotype displays characteristic behavior of stem/progenitor cell and EMT features showing high invasive ability and high expression of ZEB1 and ZEB2 [65]. Naturally occurring agents such as Garcinol (extracts from* Garcinia indica*) targeting the EMT pathways could function by downregulating ZEB1 and ZEB2 leading to E-cadherin upregulation [66].

## 7. Cervical Cancer 

In normal cervix, E-cadherin expression is found on the cell membrane of the basal and parabasal cells [67]. Loss of E-cadherin is linked with the high-risk human papillomaviruses early oncoproteins E5 [68]. Forced expression of E-cadherin in the keratinocyte cell line immortalized with HPV-16 E6 and E7 proteins could reverse the invasive phenotype [69]. The E-cadherin gene is subjected to aberrant DNA hypermethylation and the hypermethylated DNA is detectable in serum of cervical cancer patients with high risk for relapse [70]. E-cadherin expression in cervical cancer could be reactivated using HDAC inhibitor valproic acid (VPA) suggesting that histone modification and chromatin remodeling are involved in the regulation of E-cadherin in cervical cancers [71]. In the widely used cervical cancer model Hela, E-cadherin expression is undetectable [72]. Expression analysis shows that the loss and the resulting migration property are regulated by ZEB1 [73]. Low-dose radiation treatment will suppress E-cadherin expression in cervical cancer cell lines [74]. Although hypoxic has been suggested to be involved in E-cadherin suppression in solid tumors, the oxygenation status (measured by microelectrodes) has no direct correlation with the tumor E-cadherin levels in the squamous cell carcinoma of uterine cervix [75]. At present, whether ZEB1 and ZEB2 involved in the cervical cancers remained to be explored in further details. Clinically, ZEB1 expression was found in over 95% cervical cancer and the expression level was significantly associated with International Federation of Gynecology and Obstetrics (FIGO) stages and regional lymph node metastasis [67].

## 8. Colon Cancer 

The intestinal epithelium has even expression of E-cadherin in the intestinal crypts or surface epithelium [76]. E-cadherin suppression will affect the phenotypic characteristics and physiological state of colon cancer cells by reducing cell-cell adhesiveness [77]. Targeting E-cadherin inhibits glandular differentiation accounting for the undifferentiated phenotype [78]. Poorly differentiated colon cancer cells with E-cadherin expression will have an epithelial-like morphology, elevated Ca^2+^-dependent cell-cell aggregation, increased cell adhesiveness, and reduced cell motility [79]. In adenocarcinoma, there is an about 2-fold reduction in the E-cadherin transcript level in comparison with the normal colon tissues [80]. Soluble E-cadherin with the 75–85 kDa extracellular domains could be detected in the urine of colon cancer patients [81]. The association between ZEB1 with E-cadherin expression has been reported in the colon cancer cells [82, 83].

## 9. Endometrial Cancer

The association between E-cadherin loss and the invasive endometrial cancer is demonstrated by immunohistochemical staining [84]. Loss of E-cadherin has strong association with the histological subtypes of endometrial cancer. The loss is more prevalent in poorly differentiated (International Federation of Gynecology and Obstetrics (FIGO) Grade III) uterine endometrioid adenocarcinomas in comparison with the uterine serous carcinoma [85]. It is suggested that loss of E-cadherin is an early step in endometrioid cancer metastasis and the expression patterns has strong prognostic association with overall morality, disease progression, and extrapelvic recurrence [84, 86]. The loss is partly linked with E-cadherin gene hypermethylation with higher incidence in the high stage tumor [87]. In the context of ZEB expression, ZEB1 expression is not detected in the normal endometrial epithelium [19]. Exclusive expression of ZEB1 (without ZEB2) is reported in human uterus [88]. ZEB1 expression is altered in the aggressive endometrial cancer including FIGO grade 3 endometrioid adenocarcinomas, uterine serous carcinomas, and malignant mixed Müllerian tumors [89]. In differentiated Ishikawa cell line, increased ZEB1 expression could trigger the development of migratory phenotype [89]. In mouse uterine stroma and myometrium, ZEB1 protein upregulation is partly controlled by estrogen and progesterone. In the estrogen-treated mouse uterus, colocalization of ER and ZEB1 is observed [19]. Based on the expression patterns of ZEB1 in human endometrial biopsies collected at menstrual cycle with high proliferation rate, it is postulated that estrogen and progesterone could control the ZEB1 expression in human myometrial cells [19].

## 10. Gastric Cancer 

Alteration of E-cadherin gene expression is common in gastric cancers. Reduced/loss of E-cadherin expression could be caused by promoter hypermethylation induced by the microaerophilic gram-negative bacteria,* Helicobacter pylori* [90–92].* Helicobacter pylori* induced E-cadherin hypermethylation could be reversed if the bacteria are eradicated with antibiotics in the early stages [92, 93].* Helicobacter pylori* can induce the mesenchymal phenotype in gastric epithelial cell lines after 24 h in contact with elongated phenotype and loosen intercellular junctions [94]. Further, ZEB1 transcripts were increased and the corresponding protein was accumulated in the nucleus when the gastric epithelial cells are in contact with the wild type* Helicobacter pylori* [94]. ZEB1 expression level was correlated with the mesenchymal phenotype displayed by the gastric cancer [5]. In comparison with other EMT markers including Snail-1 and vimentin, aberrant expression of ZEB1 is more common in gastric cancers [95]. In human gastric cell lines, treatment with ZEB1 siRNA could effectively abrogate the mobility of cancer cells [5]. Strong expression correlation between ZEB2 and E-cadherin mRNA has been demonstrated in gastric carcinoma [96]. Gastric cancer stem cell will express a specific surface marker CD44. CD44 expression is absent in the normal epithelium and the expression will increase when the cancer progress into advanced stages [95]. CD44 expression was correlated with ZEB1 expression and was inversely correlated with the E-cadherin levels in the gastric cancer [95]. Apart from* Helicobacter pylori*, the nicotine in tobacco could also induce E-cadherin suppression by upregulating ZEB1 through the alpha7 nicotinic acetylcholine receptor in gastric cancer cells [97]. Further, continuous exposure to the low-oxygen environment could also be a contributing factor in ZEB1 and ZEB2 upregulation in gastric cancer [98].

## 11. Head and Neck Cancers

In head and neck cancers, loss of cell-cell adhesion resulting in stromal and vascular invasion as a consequence of E-cadherin dysregulation is well documented [99]. Loss of E-cadherin is common in the tumor borders in comparison with the tumor center [100]. In head and neck cancer cell lines, reduced E-cadherin expression will lead to the loss of epithelioid cell morphology [101]. E-cadherin expression is suppressed in laryngeal carcinoma, especially in supraglottic carcinoma, with significant association to poor differentiation, nodal metastasis, and advanced clinical stages [102, 103]. E-cadherin is suggested to be useful in identifying false clinically negative nodes (occult metastases) in laryngeal carcinoma patients [104]. E-cadherin could be suppressed by DNA hypermethylation or the oncoprotein expressed by the human papilloma virus [105, 106]. In addition, the loss is possibly linked with the inflammation response. Treatment with proinflammatory mediator Interleukin-1*β* on the head and neck squamous cell carcinoma cell lines will promote ZEB1 binding to the promoter region of E-cadherin [107]. The expression level of ZEB2 is correlated with delayed neck metastasis in stage I/II tongue squamous cell carcinoma patients [108].

Undifferentiated nasopharyngeal carcinoma is a unique head and neck cancer with extremely high sensitivity to ionizing radiation. Hence, radiotherapy is the first line treatment for the primary NPC patients especially when the cancer is still in the early stages. However, it is also noticed that ionizing radiation treatment may promote residual cancer migration and invasion by controlling E-cadherin expression [109]. Cancer cells with low E-cadherin level tend to be resistant to the radiation with higher clonogenic survival rate after exposing to *γ*-irradiation [109]. Further, E-cadherin loss is associated with the heterogeneous tumor microenvironment. Under hypoxic condition, E-cadherin expression is suppressed. The suppression was reversible upon oxygenation [109]. Suppressing E-cadherin expression by increasing ZEB1 expression using AKT inhibitor GSK690693 could enhance the sensitive of nasopharyngeal carcinoma cells to ionizing radiation [110].

## 12. Liver and Pancreatic Cancer

In mouse liver cancer models, loss of E-cadherin will result in metastasis [111]. Downregulation of E-cadherin could induce migration and promote EMT in liver cancer and pancreatic ductal adenocarcinoma [112, 113]. In human liver cancer, E-cadherin repression is more common in poorly differentiated cases with increased intrahepatic metastasis and poor prognosis [114]. E-cadherin suppression could be induced by the hepatitis C virus via the induced expression of osteopontin [115]. The tumor suppressing effects of E-cadherin are illustrated in liver-specific E-cadherin knockout mice. E-cadherin knockout mice will develop spontaneous liver cancer and the loss will promote chemical induced (with diethylnitrosamine) liver cancer with strong expression of stem cell marker CD44 and EMT marker vimentin [116]. Upregulation of ZEB1 is associated with thrombomodulin, a cell surface-expressed glycoprotein that is involved in inflammation and thrombosis and Claudin-1, an integral membrane protein [117, 118]. In addition, the tumor suppressor p53 could suppress ZEB1 and ZEB2 expression in the liver cancer cell lines by controlling their target microRNA expression [119]. Increase in ZEB1 expression is associated with the advanced TNM stages, intrahepatic metastasis, vascular invasion, and frequent early recurrence [120]. The inverse correlation between ZEB1 and E-cadherin has been reported in metastatic liver cancer cell lines and pancreatic tumor cell lines [113]. In pancreatic cancer, E-cadherin suppression is significantly correlated with ZEB1 and ZEB2 expression level and poor prognosis [121].

## 13. Lung Cancer

In lung cancer, genetic mutation of E-cadherin is the primary reason for E-cadherin inactivation [122]. Loss of E-cadherin is associated with the differentiation status and regional lymph node status [123, 124]. Activation of nuclear factor-*κ*B (NF-*κ*B) signaling pathways is an important regulation mechanism for E-cadherin expression in lung cancers. In alveolar type II epithelial carcinoma cell line, regulation of E-cadherin expression is partly controlled by Tank-binding kinase-1 (TBK1), inhibitor *κ*B (I*κ*B) kinase-related kinase, through activating NF-*κ*B [125]. Knocking down E-cadherin in non-small cell lung cancer cells will activate the epidermal growth factor receptor (EGFR)-MEK/ERK signaling cascade, which subsequently induce matrix metalloproteinase 2 expressions [126]. Apart from transcription regulation, it has been reported that the non-small cell lung cancer cell aberrantly expressed a misspliced (exon 11) E-cadherin transcript which was rapidly degraded by the nonsense mediated decay pathway [127]. In addition, epigenetic modification of the E-cadherin genes including DNA methylation and histone modification has been implicated in E-cadherin expression. Treatment of lung cancer cells with histone deacetylase inhibitor will inhibit the suppressing function by hindering the binding to the target sequence [128, 129]. The E-cadherin levels could be restored with the use of HDAC inhibitor Trichostatin A (7-[4-(dimethylamino)phenyl]-N-hydroxy-4,6-dimethyl-7-oxohepta-2,4-dienamide) or DNMT inhibitor 5′-Aza-deoxycytidine and the effects are partly linked with the suppression of ZEB1 in the non-small cell lung cancers [130]. ZEB1 could inhibit E-cadherin expression by recruiting histone deacetylases to the promoter regions [131]. ZEB1 upregulation in lung cancer could be controlled by cyclooxygenase-2 [132]. The expression level of E-cadherin and ZEB1 is a useful indicator of cancer cell sensitive to target therapy including epidermal growth factor receptor (EGFR) tyrosine kinase inhibitors, gefitinib, and erlotinib [131]. In addition, ZEB1 is also involved in the radiation-induced epithelial-mesenchymal transition [125]. ZEB1 expression level could also be a predictor to therapeutic responses such as resistance to epidermal growth factor receptor inhibitors for lung cancers [128].

## 14. Conclusions

The exact timing of ZEB1 and ZEB2 upregulation during malignant transformation is not clear yet. It is evidenced that ZEB1 and ZEB2 expression is induced by a sudden changes in the tumor microenvironment such as varying oxygen tensions, exposing to ionizing radiation, contacting with chemotherapeutic agents, and or demethylating agents. In several virus-associated cancers, it was found that ZEB1 and ZEB2 expression is controlled by the viral oncoproteins. In view of the fact that E-cadherin expression could counteract the migratory or invasive property of cancer cells, treatment methods targeting the suppressing mechanisms and triggering the reexpression of E-cadherin are potentially useful in controlling regional and distant metastasis. Hence, molecular dissection of the underlying mechanisms and the pathological consequence of ZEB protein upregulation in E-cadherin suppression will be useful in ameliorating theses effects in the future.
